# Enoxaparin Effect on Pregnancy Outcomes in a Patient with Elevated Plasminogen Activator Inhibitor-1

**DOI:** 10.1155/2020/7860324

**Published:** 2020-03-09

**Authors:** Kathleen Minor, Heidi Leftwich

**Affiliations:** UMASS Memorial Medical Center, 119 Belmont Street, Worcester, MA 01605, USA

## Abstract

**Background:**

Plasminogen activator inhibitor-1 (PAI-1) inhibits tPA and creates a prothrombotic state. Gene polymorphisms of PAI-1 are associated with elevated levels and adverse pregnancy outcomes.

**Case:**

A 36-year-old gravida 6, para 1-1-3-1 with elevated prepregnancy PAI-1 levels, a history of early-onset preeclampsia with severe features superimposed on chronic hypertension, intrauterine growth restriction (IUGR), and recurrent pregnancy loss (RPL), presented with a dichorionic-diamniotic twin gestation. She was managed with aspirin and enoxaparin and delivered appropriately grown twins at 36 weeks and 3 days, due to the development of preeclampsia superimposed on chronic hypertension. She was discharged not on enoxaparin and represented with pulmonary edema on postoperative day 8.

**Conclusion:**

It is reasonable to consider testing certain patients with recurrent pregnancy loss and/or early preeclampsia with severe features for PAI-1. If levels are elevated, treatment with prophylactic enoxaparin may be beneficial. Further research is needed to determine the effect of this therapy in patients with exceedingly poor perinatal outcomes to better assess for any impact on improved outcomes.

## 1. Introduction

Pregnancy necessitates an equilibrium between fibrinolysis and thrombosis, which is dynamic through gestation to maximize coagulation in the 3rd trimester [1]. During placentation and implantation, proteolysis allows for proper trophoblastic invasion and remodeling of the spiral arteries and must be maintained in balance by antifibrinolytic factors [1–4]. Plasminogen activator inhibitor-1 (PAI-1) is a major component in regulating the fibrinolytic pathway by inhibiting tissue plasminogen activator (tPA), which normally cleaves plasminogen to plasmin and in turn cleaves fibrin into degradation products. Thus, when tPA is inactivated by PAI-1, a prothrombotic state is established [1–4]. PAI-1 is released from endothelial cells, activated platelets, and both trophoblasts and placental vasculature during pregnancy [1, 2, 4]. Progesterone increases the expression of PAI-1 from the decidua and the release of PAI-2 exclusively from the placenta as a component of systemic upregulation of thrombotic factors in pregnancy [5]. Thus, PAI-1 is a marker of endothelial dysfunction, whereas PAI-2 is often utilized as a surrogate for placental function [2, 4]. In healthy gravid women, PAI-1 levels increase by 4-5-fold in the third trimester and return to normal levels after delivery [6].

The PAI-1 gene promoter region has well-characterized polymorphisms, specifically the 4G insertion/5G deletion polymorphism, which enhances transcription of PAI-1 [7, 8]. The expression of PAI-1 is modified by atherosclerotic risk factors that induce endothelial damage, including diabetes, insulin resistance, and hypertriglyceridemia [8]. These risk factors can cause local hypoxia, which alters cell-type specific expression of PAI-1 [3, 8]. There is a controversial association between PAI-1 polymorphisms and adverse pregnancy outcomes, such as the risk of preeclampsia, intrauterine growth restriction (IUGR), and recurrent pregnancy loss (RPL).

Early-onset preeclampsia is disproportionally responsible for perinatal morbidity and mortality [4]. Preeclampsia has a spectrum of phenotypic presentations with divergent onset and severity, with likely more than one etiology underlying the disease, each converging on a central process of abnormal activation of coagulation, fibrinolysis, and inflammation [2, 3, 6]. The inciting event with early-onset disease is believed to occur during placentation, with dysfunctional invasion of the trophoblasts and remodeling of spiral arteries, leading to uteroplacental ischemia, intravascular inflammation, and endothelial dysfunction [1, 2, 4]. Preexisting inherited or acquired thrombophilia may predispose to this abnormal placentation [4]. Further evidence of an underlying hypercoagulable state is demonstrated in pathologic studies of placentas from patients with preeclampsia demonstrating villous infarcts, fibrin deposition, and thrombosis [3, 4].

Despite a mechanistic pathway and biologic plausibility of increased PAI-1 levels leading to preeclampsia and RPL, testing for PAI-1 is not currently recommended. Additionally, there is a paucity of data regarding treatment modalities if elevated levels are found. We present a case that enhances our understanding of risk factors for preeclampsia and elucidates a possible treatment strategy with enoxaparin for those with preexisting elevated PAI-1 levels. Informed consent was obtained.

## 2. Case

A 36-year-old gravida 6, para 1-1-3-1 nonsmoker with a history of chronic hypertension not on antihypertensive medication, migraines, obesity with a BMI of 43 kg/m^2^, and asthma, presented at 8-week gestation with dichorionic-diamniotic twins. The gestation was conceived after clomid and intrauterine insemination (IUI) with donor sperm, as she did not have a current partner. Her first pregnancy was a miscarriage at 6-week gestation after IUI using a unique sperm donor than subsequent pregnancies. Gravida 2 was conceived via IUI and was complicated by a vanishing twin and development of preeclampsia with severe features (pulmonary edema), necessitating a primary lower transverse cesarean section at 31 weeks and 4 days gestational age. Placental pathology demonstrated marked villous hypermaturity, nontransformed decidual vessels, and multiple placental infarcts. Gravida 3 was conceived via IUI and was complicated by the diagnosis of VACTERL syndrome. She was managed on low-dose aspirin and eventually labetalol during this gestation. She underwent a repeat cesarean at 37 weeks for worsening chronic hypertension and IUGR, with placental pathology showing placental infarction and a single umbilical artery. Her blood pressure was significantly elevated during this time; however, she was not considered to have preeclampsia by the delivering team and magnesium was not initiated. On day of life 5, there was a neonatal demise due to surgical complications from a bowel obstruction. Gravidas 4 and 5 were early miscarriages. Her family history was relevant for a mother who had two miscarriages and cardiovascular disease.

She underwent preconception consultation with hematology. She was tested for inherited thrombophilias and found to have an increased PAI-1 antigen level to 64 ng/mL (normal 4-43 ng/mL). Testing for Factor V Leiden mutation, Prothrombin Gene mutation, Protein C, Protein S, and Antithrombin III deficiency were negative. Additional workup included normal antiphospholipid antibody syndrome testing, thyroid studies, hemoglobin A1c, and hysterosalpingogram. Due to her elevated PAI-1 and history of poor perinatal outcomes, prophylactic enoxaparin was suggested by hematology. She was maintained on aspirin 81 mg starting at 12 weeks and enoxaparin 40 mg subcutaneously daily starting at the time of her positive pregnancy test after significant discussion with maternal-fetal medicine regarding her history and the hematology consultation. She had normal baseline kidney function, liver function, and no chronic proteinuria. Her antenatal course was uncomplicated apart from presenting at 31 weeks with a headache and elevated blood pressure. Labs ruled her out for preeclampsia; she was given betamethasone, started on labetalol 100 mg twice daily, and followed with weekly labs. Her headache improved and was noted to be consistent with her baseline migraines. She underwent repeat cesarean at 36 weeks and 3 days due to preeclampsia without severe features superimposed on chronic hypertension. Infants weighed 3,046 g and 2,630 g. Interestingly, placental pathology showed a mature dichorionic diamniotic twin placenta, with no vasculopathy and no placental infarction. She received enoxaparin postpartum in the hospital, in the setting of her cesarean section and obesity, but declined to continue treatment for 6 weeks postpartum. She represented on postpartum day 8 with severe range blood pressures and dyspnea with imaging confirming bilateral pulmonary edema. An echocardiogram revealed a normal ejection fraction of 65%. Her labetalol was increased to 400 mg twice daily and she was discharged home on Lasix 20 mg twice daily for 5 days, with full and rapid recovery. She was titrated off labetalol in the subsequent weeks and remained off antihypertensive medications. In context of chronic hypertension and preeclampsia, it was suggested that she could consider continuing aspirin for secondary prevention of cardiovascular disease.

## 3. Discussion

In 1984, Wiman et al. was the first to characterize the association between increased PAI-1 levels and preeclampsia [8]. A meta-analysis of 6 case control studies investigating the 4G/5G polymorphism found no significant difference in the frequency of the 4G allele in patients with and without preeclampsia; however, patients with the 4G allele had a 1.27-fold increased risk of preeclampsia [7, 8]. Similarly, a meta-analysis of 18 case control studies found that the 4G/5G polymorphism is associated with an increased risk of RPL (OR = 1.7, 95%CI = 1.21 − 2.38), and advocated for PAI-1 screening and treatment with anticoagulation [7].

Studies investigating PAI-1 levels in women with preeclampsia have conflicting results: some showing no difference [1], whereas other studies demonstrate a significantly higher maternal concentration of PAI-1 in women with preeclampsia compared to low risk pregnant women [6, 9, 10]. Wilkstrom et al. demonstrated that the ratio of PAI-1 to PAI-2 was increased in early-onset preeclampsia (defined as 24-32-week gestation), but not late-onset disease [2, 10]. The ratio increased prior to clinical symptoms, indicating it may have predictive value [9, 10]. This points to a possible confounder in studies, which combine patients with early- and late-onset preeclampsia [2, 4, 10].

Evidence is lacking for treatment with anticoagulation to prevent adverse pregnancy outcomes in women with inherited thrombophilia [11]. One study investigated the effect of low molecular weight heparin (LMWH) in 50 women with previous adverse pregnancy outcomes and a known “novel” thrombophilia (which include those with PAI-1 polymorphism) versus a conventional thrombophilia. It concluded that LMWH reduced the incidence of growth restriction, IUFD, and preterm birth. Additionally, treatment was more beneficial with novel than conventional thrombophilia [12].

We present a case that we believe provides justification for future research to prove benefit of enoxaparin in patients with elevated PAI-1 levels. Our patient had elevated prepregnancy PAI-1 levels, a history of RPL, IUGR, and early-onset preeclampsia, managed on enoxaparin and aspirin during pregnancy. Despite new risk factors in this most recent gestation of AMA, a twin gestation, obesity, and chronic hypertension, she had delayed onset of preeclampsia, appropriately grown twins and no pathognomonic findings of disease on placental pathology. She did develop pulmonary edema 8 days postpartum off anticoagulation, signifying continuation of anticoagulation for 6 weeks postpartum may be warranted. Enoxaparin was the only new management strategy in this pregnancy compared to her prior gestations. Although there are confounding factors including obesity, chronic hypertension, and use of ART, this case suggests that the use of enoxaparin and aspirin may be an appropriate strategy in women with elevated PAI-1 levels who have additional risk factors for adverse pregnancy outcomes. Baby aspirin was initiated using the 81 mg daily dosing, which is the standard of care for high-risk patients in the United States, as this was the location of this case report. However, the authors are aware of European data suggesting aspirin has a dose-dependent response and that when utilized in higher doses, may have a more profound effect on the decrease in risk. Should the higher dose of baby aspirin have been recommended, dose and treatment with enoxaparin may have been reconsidered.

The implication of this therapy on early-onset preeclampsia and IUGR is an exciting direction of future research. Further randomized controlled studies are needed to identify the cohort of patients with adverse pregnancy outcomes who would benefit from testing for PAI-1. If therapy is to be considered, a true understanding of the adverse events related to treatment and a cost analysis on a population basis need to be investigated. Additionally, the effect of anticoagulation with enoxaparin versus antiplatelet agents needs to be isolated.

Regardless, our patient with multiple new risk factors for preeclampsia, compounded by her prior risk factors, had a successful 36-week gestation with a healthy placenta and appropriate neonatal weights. We propose that screening for PAI-1 and discussion regarding anticoagulation could be performed in women at highest risk for adverse pregnancy outcomes, with otherwise negative thrombophilia screening. If anticoagulation is to be considered, treatment specifics must be elucidated including whether the standard prophylactic or weight adjusted dose is preferred and the optimal timing of initiation and duration.
